# Treg/Th17 imbalance and its clinical significance in patients with hepatitis B-associated liver cirrhosis

**DOI:** 10.1186/s13000-019-0891-4

**Published:** 2019-10-21

**Authors:** Yong-Ting Lan, Zhen-li Wang, Peng Tian, Xiao-Na Gong, Yu-Chen Fan, Kai Wang

**Affiliations:** 1 Department of Hepatology, Qilu Hospital of Shandong University and Institute of Hepatology, Shandong University, Wenhuaxi Road 107#, Jinan, 250012 People’s Republic of China; 2 Department of Gastroenterology, Zibo Central Hospital, Zibo, Shandong People’s Republic of China; 3 Department of Hepatology, Qing Dao No. 6 People’s Hospital, Qingdao, Shandong People’s Republic of China; 4 Institute of Hepatology, Shandong University, Jinan, Shandong People’s Republic of China

**Keywords:** Hepatitis B-associated liver cirrhosis, Immune mechanism, Treg, Th17

## Abstract

**Background:**

Recent studies have shown that T cell-mediated cellular immune mechanisms play important roles in the progression of hepatitis B to liver cirrhosis, but the underlying mechanisms remain unclear. This present study was aimed to determine the relationship between Treg/Th17 and hepatitis B-associated liver cirrhosis.

**Methods:**

The Treg and Th17 cell frequencies in the peripheral blood of all participants, including 93 patients with hepatitis B-associated liver cirrhosis and 40 healthy subjects, were measured by flow cytometer. Cox regression model and receiver operating characteristic(ROC) curves were applied to investigate the prognostic significance of Treg/Th17 ratio in decompensated liver cirrhosis.

**Results:**

We observed the Treg/Th17 imbalance was present in patients with hepatitis B-associated liver cirrhosis, with reduced Treg cells in their peripheral blood, increased Th17 cells and decreased Treg/Th17 ratio. Treg and Th17 cells were negatively correlated. Treg/Th17 imbalance was closely related to the clinical stage of hepatitis B-associated liver cirrhosis. The Virus load, Treg frequencies and the Treg/Th17 ratio were independent factors predicting decompensated liver cirrhosis from a Cox regression model. The ROC analysis showed that the Treg/Th17 ratio was the best marker for predicting decompensated liver cirrhosis.

**Conclusions:**

Treg/Th17 imbalance is involved in the pathogenesis of hepatitis B-associated liver cirrhosis and the Treg/Th17 ratio can be used as a potential marker for predicting decompensated liver cirrhosis.

## Introduction

Hepatitis B virus (HBV) is a hepatotropic DNA virus that has infected 350 million people globally. Approximately 2–6% of untreated patients with chronic hepatitis B virus infection progress to hepatitis B-associated liver cirrhosis each year [1]. Liver cirrhosis is the final stage of liver fibrosis and can be divided into compensated liver cirrhosis (CLC) and decompensated liver cirrhosis (DCLC) according to the Child-Pugh classification. The 5-year survival rate of patients with compensated liver cirrhosis is approximately 55%, whereas that of patients with decompensated liver cirrhosis drops significantly to approximately 14% due to frequent complications such as gastrointestinal bleeding, hepatic encephalopathy, and peritoneal ascites. Therefore, the ability to delay the progression of liver fibrosis and prevent its progression from the compensated phase to decompensated phase through medication has become the key to improving the survival rate of patients with liver cirrhosis. At present, anti-hepatic fibrosis treatment is mainly based on antiviral therapy through a mechanism of effective prevention of hepatitis virus replication and alleviation of persistent hepatic injuries to promote the repair of liver tissue and delay the progression of liver cirrhosis. However, side effects during treatment, such as virus mutations and drug resistance, seriously affect the efficacy of the drugs. Therefore, exploration of new pathogenesis mechanisms of hepatitis B-associated liver cirrhosis to find anti-liver fibrosis drugs with better efficacy and fewer side effects is a new direction of current research.

The latest research has shown that the pathogenic mechanism underlying the progression of hepatitis B to liver cirrhosis is mainly the result of the combined actions of the virus and the body’s cellular immune response following hepatitis B virus infection, with T lymphocytes playing a vital role in the immune response to persistent HBV infection [2]. Therefore, it is especially important to investigate the relationship between the T cell-mediated immune response and the pathogenic mechanism of hepatitis B-associated liver cirrhosis. Helper T cells (Th) are important components of the immune system and produce a variety of cytokines that participate in the body’s anti-viral immune response and perform functions crucial for viral clearance and the progression of liver fibrosis. According to their biological characteristics, helper T cells are divided into four types: Th1, Th2, Th17 and Treg cells. In the past, studies of hepatic fibrosis focused on the two classical T cell subtypes, Th1 and Th2. The Th1/Th2 balance plays an important role in maintaining normal human body function. This model also explains the pathogenesis of liver fibrosis in some cases. However, recent progress in immunology has identified two new T cell subsets (Treg and Th17 cells) that complement the classical Th1/Th2 model [3] .

A close, complex relationship exists between Th17 and Treg cells. Th17 cells mediate the inflammatory response, while Tregs mediate immune tolerance. However, Th17 and Treg cells have a common origin, naive T cells. Although most of the chemotactic receptors on the surface of Th17 and Treg cells are the same [4], their functions and differentiation processes are antagonistic to each other. Under normal circumstances, Th17 and Treg cells maintain a dynamic balance and result in the induction of immune responses of appropriate intensity, which is conducive to the maintenance of a stable immune state in the body. Treg/Th17 imbalance is closely related to many immune disorders, tumors, and infectious diseases [5] .

Current studies have confirmed that Treg/Th17 imbalance is present in patients with chronic hepatitis B. Th17 and related cytokines participate in specific immune responses to HBV that promote inflammation and have potential antiviral effects. In addition to inhibiting an excessive immune response and alleviating liver inflammation, Tregs also indirectly lead to chronicity of viral infection. Therefore, dysregulation of Treg/Th17 balance causes persistent HBV infection and liver injuries from inflammation [6]. Nan et al. [7] found elevated levels of Th17 and Treg cells in the peripheral blood of patients with chronic hepatitis B, and the increase in Th17 cells was more evident during acute episodes, which led to a reduced Treg/Th17 ratio. This finding suggests that Treg/Th17 imbalance is closely associated with the progression of chronic hepatitis B. However, there is a lack of systematic studies on the changes in Treg/Th17 in patients with hepatitis B-associated liver cirrhosis. Therefore, this study examined the significance of Treg/Th17 imbalance in the development and progression of hepatitis B-associated liver cirrhosis through examination of peripheral Treg and Th17 counts in patients with hepatitis B-associated liver cirrhosis to provide new targets for clinical treatment of hepatitis B-associated liver cirrhosis.

## Methods

### Study population

A total of 93 patients with hepatitis B-associated liver cirrhosis who had never been treated before seeking treatment at Qilu Hospital of Shandong University between January 2016 and June 2016 were selected as the liver cirrhosis (LC) group. All cases were diagnosed according to the “Guidelines for the Prevention and Treatment of Chronic Hepatitis B” jointly formulated by the Chinese Society of Hepatology and the Society of Infectious Diseases of Chinese Medical Association in 2015; patients with hepatocellular carcinoma,other viral hepatitis infections, alcoholic hepatitis, autoimmune hepatitis, other non-viral hepatitis, or other serious systemic diseases were excluded. According to the Child-Pugh classification, 45 patients were classified into the CLC group (Child-Pugh grade A), 48 patients were classified into the DCLC group (Child-Pugh grades B and C), and 40 healthy subjects were enrolled into the control group. All enrolled subjects signed the informed consent. This study was approved by the Medical Ethics Committee of Shandong University and strictly adhered to all the ethical principles of medical research involving human subjects in the Helsinki Declaration.

### Collection of clinical data

Serum samples were collected after the participants had fasted for at least 8 h. The serum biochemical markers of liver function that were assessed in this study using an automatic biochemical analyzer (Cobas c311, Roche Diagnostic Ltd., Germany) included alanine aminotransferase (ALT), total bilirubin (TBIL), and albumin (ALB). Prothrombin time (PT) was evaluated with an automatic coagulometer (Stago, Inc., France). The virus load was measured with a real-time PCR machine (Life Technologies 7500, Life Technologies Co, Ltd., USA).

### Flow cytometry analysis

All products were purchased from eBiosciences, an Affymetrix company (San Diego, CA, USA). In total, 5 mL of fasting venous peripheral whole blood samples was collected from study subjects in the morning in heparin-treated tubes. To detect Treg cells, PBMCs without stimulation were surface stained with the eZFluorTM anti-human CD4-FITC and CD25-APC cocktail, followed by treatment with a working fixation/permeabilization solution, permeabilization buffer, and normal rat serum. Intracellular staining was performed with anti-human Foxp3-PE or rat IgG2a K isotype control-PE according to the manufacturer’s instructions. To evaluate Th17 cells, PBMCs were stimulated with Cell Stimulation Cocktail in complete culture medium (RPMI 1640 supplemented with 10% FBS and 200 mM L-Gln) for 5 h at 37 °C in 5% CO2. Then, the cells were incubated with anti-human CD4-FITC or mouse IgG1 K isotype control-FITC, followed by a working fixation/permeabilization solution and permeabilization buffer. Intracellular staining was performed with anti-human IL-17A-PE or mouse IgG1 K isotype control-PE according to the manufacturer’s instructions. The flow cytometry analyses were performed using a FACSCalibur flow cytometer (BD Biosciences, USA), and the data analysis was conducted using CellQuest software (BD Biosciences, Franklin Lakes, NJ, USA).

### Statistical analysis

With GraphPad Statmate 2.0, the minimum sample size in present study was 51 samples (α = 0.05, β = 0.10).The data analysis was performed using GraphPad Prism 5.01 (GraphPad Software, 2007, La Jolla, CA, USA) and SPSS 19.0 (SPSS Inc., Chicago, IL, USA). The data are expressed as means ± standard deviations. The independent samples t-test was used for statistical comparisons between two groups. The correlation between Treg and Th17 cells was evaluated using Spearman’s correlation analysis. The independent factors predicting DCLC were assessed using a Cox regression model. A receiver operating characteristic (ROC) curve was analyzed to evaluate the diagnostic and prognostic values of the tested parameters. *p* < 0.05 was considered statistically significant.

## Results

### Baseline clinical characteristics

As shown in Table 1, no difference was found in gender and age between the three groups (*p* > 0.05). However, the ALT, TBIL, ALB, and PT levels and virus loads significantly differed at enrollment (*p* < 0.05).

**Table 1:** Clinico-pathological information of participants

Variables	Ctrl	CLC	DCLC	*p* value
Case(n)	40	45	48	
Gender(M/F)	27/13	31/14	33/15	0.2167
Age(years)	44.2 ± 13.4	45.2 ± 12.9	47.6 ± 15.6	0.1635
ALT(U/L)	33.6 ± 7.9	43.6 ± 10.3	185.1 ± 27.1	0.0006*
TBIL(μmol/L)	10.3 ± 4.9	25.2 ± 11.9	77.4 ± 22.1	0.0009*
ALB(g/L)	37.6 ± 2.2	36.3 ± 3.1	28.3 ± 5.3	0.0052*
PT(s)	12.3 ± 2.1	14.3 ± 1.4	18.9 ± 2.8	0.0076*
Virus load (IU/ml)	–	5.2 ± 1.2	6.3 ± 0.9	0.0082*

*Indicates a significant difference (*p* < 0.05)

### Decreased Tregs, increased Th17 cells, and reduced Treg/Th17 ratio in the peripheral blood mononuclear cells of the LC group

**Fig. 1:**
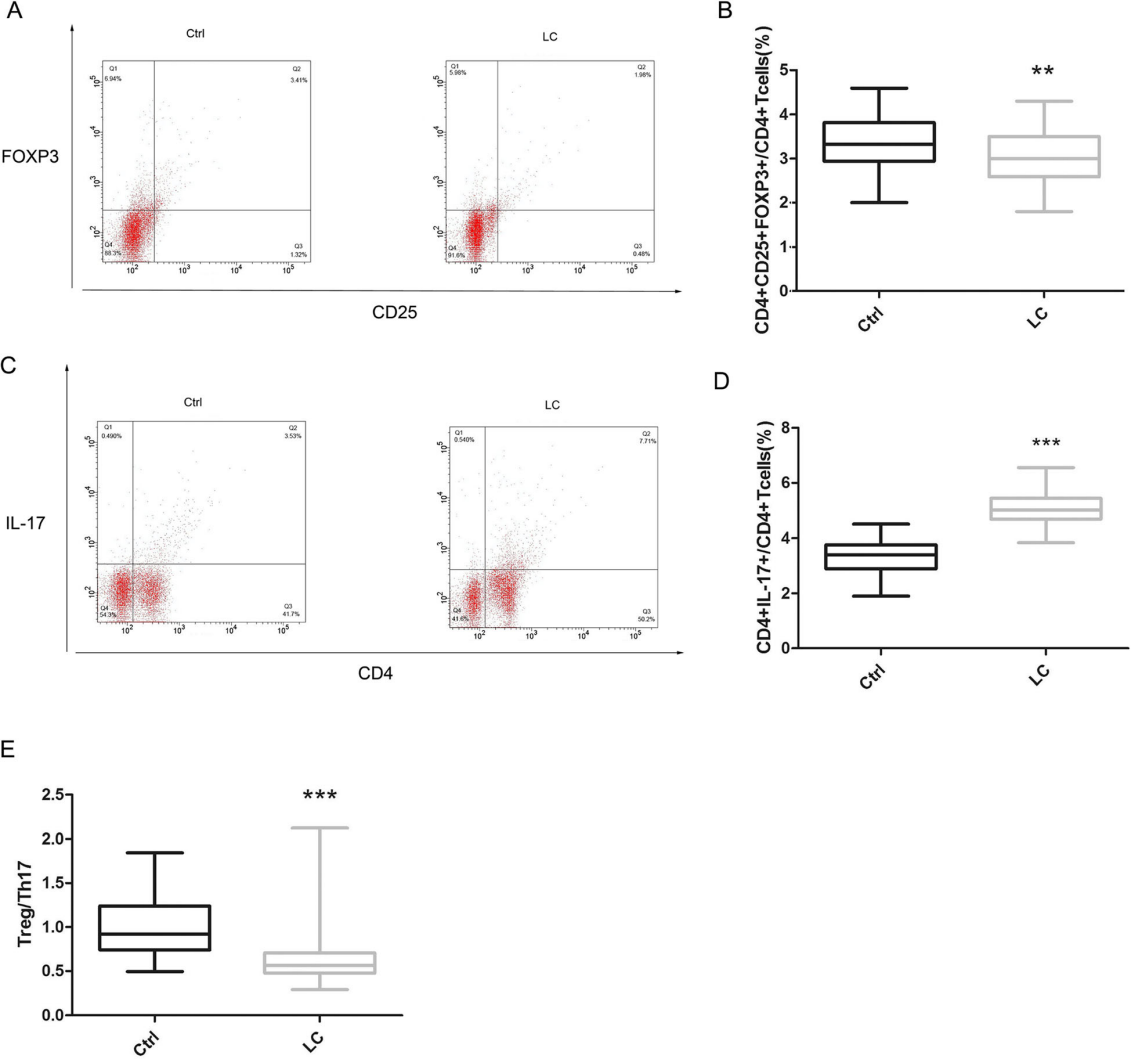
Comparison of the incidence of Treg and Th17 cells and the Treg/Th17 ratio between the two groups. Ctrl refers to the control group, LC refers to the liver cirrhosis group. * *p* < 0.05, ** *p* < 0.01, *** *p* < 0.001. **a** Flow cytometric analysis of CD4^+^CD25^+^FOXP3^+^ Treg cells between the two groups. **b** Comparison of the percentage of CD4^+^CD25^+^FOXP3^+^ Treg cells in CD4^+^ T cells between the two groups (*p* < 0.01). **c** Flow cytometric analysis of CD4^+^IL-17^+^ Th17 cells between the two groups. **d** Comparison of the percentage of CD4^+^IL-17^+^ Th17 cells in CD4^+^ T cells between the two groups (*p* < 0.001). (E) Comparison of the Treg/Th17 ratio between the two groups (*p* < 0.001)

Compared with the control group, the LC group was found to have a significantly reduced percentage of CD4^+^CD25^+^FOXP3^+^ Treg cells (*p* < 0.01, Fig. 1a and b), markedly increased CD4^+^IL-17^+^ Th17 cells (*p* < 0.001, Fig. 1c and d), and a significantly lower Treg/Th17 ratio (*p* < 0.001, Fig. 1e).

### Decreased Tregs, increased Th17 cells, and reduced Treg/Th17 ratio in the peripheral blood mononuclear cells of the DCLC group

**Fig. 2:**
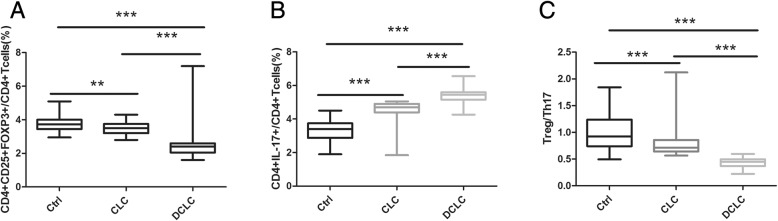
Comparison of the incidence of Treg and Th17 cells and the Treg/Th17 ratio among the three groups. Ctrl refers to the control group, CLC refers to the compensated liver cirrhosis group, DCLC refers to the decompensated liver cirrhosis group. * *p* < 0.05, ** *p* < 0.01, *** *p* < 0.001. **a** Comparison of the percentage of CD4^+^CD25^+^FOXP3^+^ Treg cells in CD4^+^ T cells among the three groups **b** Comparison of the percentage of CD4^+^IL-17^+^ Th17 cells in CD4^+^ T cells among the three groups. **c** Comparison of the Treg/Th17 ratio among the three groups

As shown in Fig. 2a, compared to the control group, both the CLC group (p < 0.01) and the DCLC group (*p* < 0.001) showed lower percentages of CD4^+^CD25^+^FOXP3^+^ Treg cells, with the percentage of CD4^+^CD25^+^FOXP3^+^ Treg cells of the DCLC group being lower than that of the CLC group (*p* < 0.001). As shown in Fig. 2b, compared to the control group, both the CLC group (p < 0.001) and the DCLC group (p < 0.001) showed higher percentages of CD4^+^IL-17^+^ Th17 cells, with the percentage of CD4^+^IL-17^+^ Th17 cells of the DCLC group being higher than that of the CLC group (*p* < 0.001). As shown in Fig. 2c, compared to the control group, both the CLC group (p < 0.001) and the DCLC group (p < 0.001) showed decreased Treg/Th17 ratios, with the Treg/Th17 ratio of the DCLC group being lower than that of the CLC group (p < 0.001).

### Treg and Th17 cells in the peripheral blood mononuclear cells of the LC group exhibited a negative correlation

**Fig. 3:**
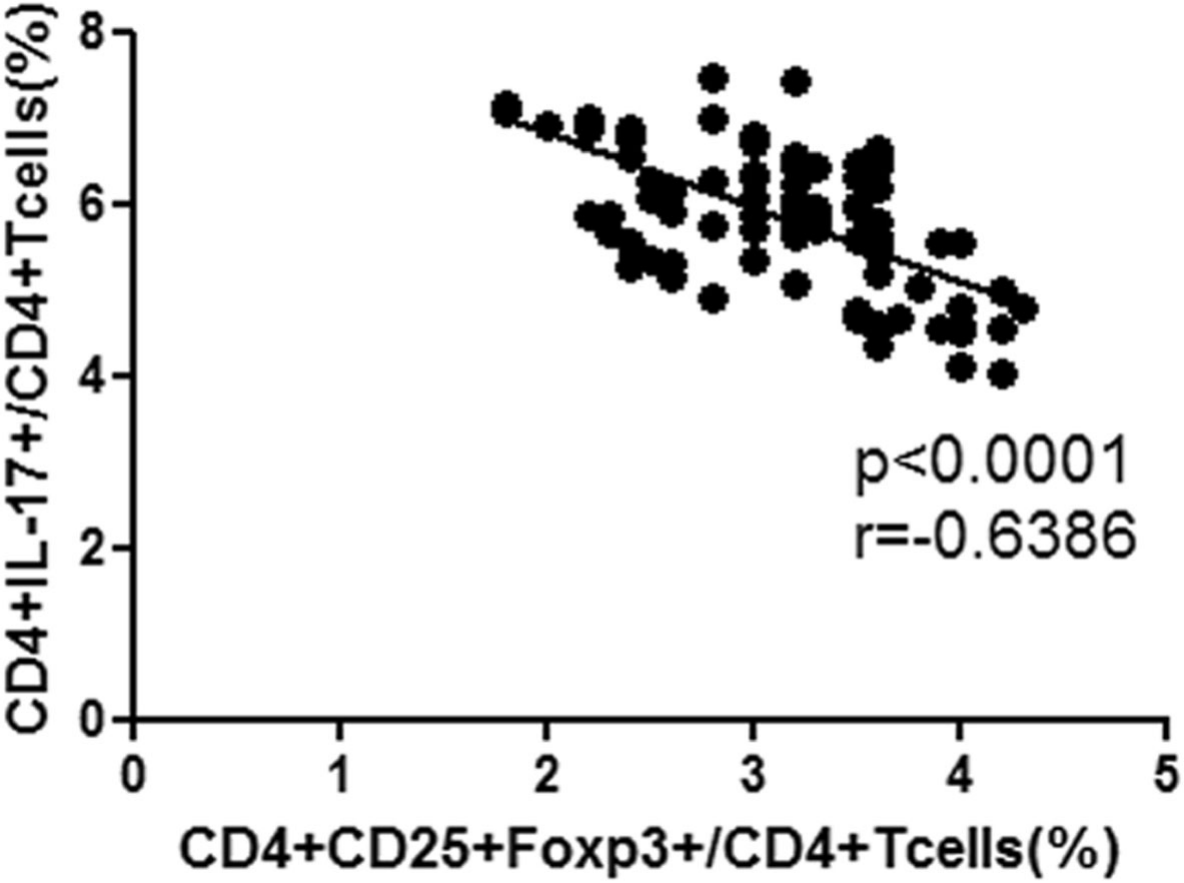
Negative correlation between Treg and Th17 cells in the peripheral mononuclear cells of the LC group (r = − 0.6386, *p* < 0.0001, n = 93)

Compared with the CLC group, the DCLC group exhibited significantly reduced Tregs (p < 0.001) and significantly markedly increased Th17 cells (*p* < 0.001). Therefore, the relationship between Treg and Th17 cells in the peripheral blood mononuclear cells from the LC group was analyzed using Spearman’s correlation analysis. The results indicated a negative correlation between Treg and Th17 cells in the peripheral blood mononuclear cells of the LC group (r = − 0.6386, *p* < 0.0001, *n* = 93; Fig. 3).

### Association of the independent factors with LC progression

Based on these results, a Cox regression model was conducted to analyze the parameters related to LC. We demonstrated that virus load,Treg frequencies and the Treg/Th17 ratio were independent factors predicting DCLC (Table 2).

**Table 2:** Cox regression analysis of the risk factors in patients of LC

Variables	Univariate			Multivariate		
	OR	95% CI	*p* value	OR	95% CI	*p* value
ALT	1.005	0.965 ∼ 1.516	0.312			
TBIL	1.017	0.998 ∼ 1.217	0.416			
ALB	0.732	0.542 ∼ 0.891	0.092			
PT	1.335	1.082 ∼ 1.679	0.243			
Virus load	1.182	1.052 ∼ 1.331	0.040	1.752	0.699 ∼ 0.882	0.032
Treg	0.832	0.550 ∼ 1.331	0.023	0.604	0.764 ∼ 0.923	0.015
Th17	1.766	1.241 ∼ 1.908	0.078			
Treg/Th17	0.652	0.120 ∼ 1.107	0.007	0.474	0.862 ∼ 0.976	0.003

### ROC analysis of the virus load, Treg cell frequencies,Th17 cell frequencies and Treg/Th17 ratio to discriminate CLC and DCLC

**Fig. 4:**
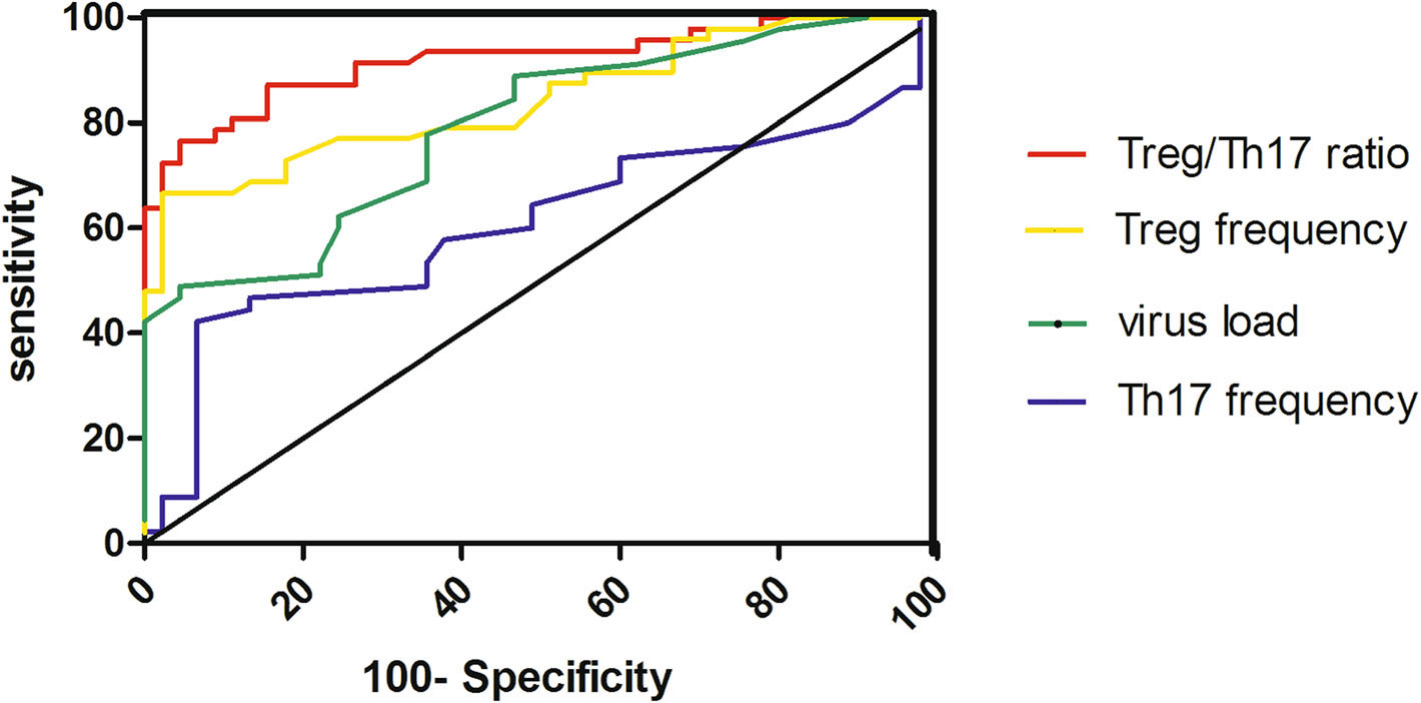
ROC curves of the virus load,Treg cell frequencies,Th17 cell frequencies and the Treg/Th17 ratio for the prediction of DCLC

We plotted ROC curves for the virus load,Treg cell frequencies,Th17 cell frequencies and the Treg/Th17 ratio for a related sensitivity analysis (Fig. 4). The area under the ROC curve of the virus load for the DCLC prediction was 0.790, showing its predictive ability (*p* < 0.001). Additionally, the area under the ROC curve of the Treg frequency was 0.844, revealing its predictive power (*p* < 0.001). However, the area under the ROC curve of the Th17 frequency was 0.608, revealing no predictive ability (*p* = 0.078). Finally, the area under the ROC curve for the Treg/Th17 ratio was 0.912, demonstrating its predictive ability (p < 0.001). From these results, we conclude that the Treg/Th17 ratio is the best indicator for predicting DCLC.

## Discussion

Liver cirrhosis is a common chronic progressive liver disease that is marked by diffuse liver damage caused by the long-term or repeated actions of one or more pathogenic factors. Among these factors, HBV is a major cause of liver cirrhosis. Liver fibrosis is an inevitable stage in the progression of chronic hepatitis B to hepatitis B-associated liver cirrhosis. It has been reported that the development of hepatitis B-associated liver cirrhosis is mainly associated with Kupffer cells and hepatic stellate cells (HSC) and that HSC activation and proliferation is the central link in the pathogenesis of liver fibrosis [8, 9]. When the liver is damaged by inflammation, HSCs located in the gap between hepatocytes and hepatic sinusoidal endothelial cells are activated [10] and synthesize a large amount of extracellular matrix [11] to promote the occurrence and progression of liver cirrhosis.

In recent years, Treg and Th17 cells have been found to have a relationship that is both synergistic and antagonistic. They function coordinately in differentiation and development but with mutual restrictions, and both are closely related to the development and progression of hepatitis B-associated liver cirrhosis. Study of the relationship between Treg and Th17 cells and hepatitis B-associated liver cirrhosis will facilitate further investigation of the pathogenesis of hepatitis B-associated liver cirrhosis and provide the basis for new strategies in the treatment of hepatitis B-associated liver cirrhosis.

Tregs comprise a T cell subset that accounts for approximately 5–10% of peripheral CD4^+^ T cells [12]. Tregs mainly express CD4, CD25 and FOXP3, where FOXP3 is a specific transcription factor in Treg cells, and thus, Treg cells are often defined as CD4^+^CD25^+^FOXP3^+^ cells [13].Under a normal physiological state, Treg cells participate in the regulation of immune tolerance in the body to maintain normal immune functions [14]. Claassen et al. [15] found a large number of Treg cells in the liver in their study on hepatic fibrosis caused by hepatitis C virus, and a larger number of Treg cells was associated with less severe liver fibrosis, suggesting that Treg cells are likely to have an inhibitory effect on the progression of liver fibrosis. In the present study, compared to the control group, the LC group showed a decreased percentage of CD4^+^CD25^+^FOXP3^+^ Treg cells (*P* < 0.01); the DCLC group had a lower percentage of CD4^+^CD25^+^FOXP3^+^ Treg cells (*P* < 0.001) than that of the CLC group, indicating that Treg cells not only participated in the pathogenesis of hepatitis B-associated liver cirrhosis but also played a positive regulatory role in preventing liver fibrosis during the course of the disease. The anti-hepatic fibrosis effect of Treg cells is likely to be related to IL-10 secretion. IL-10 is a major immunosuppressive effector secreted by Treg cells. It protects liver cells and antagonizes hepatocellular injury and hepatic fibrosis. IL-10 exerts its anti-liver fibrosis effect through the following possible mechanisms of action: (1) TGFβ1 stimulates the production of collagen fibers by HSCs, and IL-10 exerts its anti-fibrosis effect through reducing the expression of TGFβ1 [16]; (2) IL-10 suppresses or down-regulates the expression of TNFα, PDGF-β and COX-2 in liver tissues and, thus, inhibits the activation of HSCs; and (3) IL-10 inhibits the proliferation response in the acute phase of liver injury and further impedes the occurrence and development of liver fibrosis.

Th17 cells comprise another subset of CD4^+^ T cells. In contrast to the functions of Tregs, Th17 cells mediate inflammatory responses and play an important role in autoimmune diseases, tumors, and infectious diseases. Th17 cells secrete a variety of cytokines, the main of which are IL-17F, IL-17A, IL-22, and IL-21; IL-17A is considered the most characteristic cytokine [17]. IL-17 mobilizes, recruits and activates neutrophils to promote an inflammatory response and progression of autoimmune diseases [18]. Sun et al. [19] showed that the increase in Th17 cells in the liver of patients with hepatitis B-associated liver cirrhosis promoted the increase of HSC activity and eventually led to deterioration of hepatitis B-associated liver cirrhosis. In the study by Sparna et al. [20], Th17 cells in the liver were found to be mostly concentrated in areas with more severe liver fibrosis, and the number of Th17 cells was positively correlated with serological markers that reflect the degree of hepatic fibrosis, suggesting that Th17 cells may play an important role to promote the development of liver fibrosis. The results of this study are consistent with those of the above studies. Compared with the control group, the LC group showed an increased percentage of CD4^+^IL-17^+^ Th17 cells (*p* < 0.001). Moreover, the DCLC group had a greater percentage of CD4^+^IL-17^+^ Th17 cells than the CLC group (*p* < 0.001), suggesting that Th17 cells not only participated in the pathogenesis of hepatitis B-associated liver cirrhosis but also exerted a negative regulatory function to promote the development of liver fibrosis.

Th17 cells can increase the immune response mainly through the secretion of IL-17, which is likely to participate in the development and progression of liver fibrosis. The results from the study by Tan [21] showed increased IL-17A expression in patients with hepatic fibrosis caused by hepatitis B virus, which indicated that IL-17A plays an important role in the progression of liver fibrosis. Meng et al. [22] have demonstrated that IL-17 promotes liver fibrosis through two independent mechanisms: (1) IL-17 stimulates Kupffer cells (KCs), which in turn secrete the cytokines IL-6, IL-1β, TNF-α and TGF-β1, which stimulate the activation of HSCs; (2) IL-17 directly stimulates the STAT3 signaling pathway, activates HSCs to express collagen I, and promotes the activated HSCs to transform into pro-fibrogenic myofibroblasts, resulting in the synthesis of large amounts of extracellular matrix. Taking into account the role of Th17 cells in liver fibrosis, they may represent a new target for the treatment of liver fibrosis.

This study found that the LC group had a significantly decreased Treg/Th17 ratio when compared with that of the control group (*p* < 0.001), which indicated that Treg/Th17 imbalance was present in the LC group and was likely to participate in the pathogenesis of hepatitis B-associated liver cirrhosis. Further study found the Treg/Th17 ratio of the DCLC group was significantly lower than that of the CLC group (p < 0.001). The results of this study showed that Treg/Th17 imbalance is closely related to the progression of hepatitis B-associated liver cirrhosis. Additionally, we found that the ALT, TBIL, ALB, and PT levels, and the virus load significantly differed between the DCLC, CLC, and control groups (*p* < 0.05,Table 1). Further study revealed that virus load,Treg cell frequencies and the Treg/Th17 ratio were predictors of progression of hepatitis B-associated liver cirrhosis (Table 2). Finally, the ROC analysis showed that the Treg/Th17 ratio was the best indicator for predicting DCLC (Fig. 4).In a summary, the Treg/Th17 ratio can be used as an evaluation indicator for functional classification and prognosis of hepatitis B-associated liver cirrhosis. A lower Treg/Th17 ratio is expected to be associated with worse hepatic functions and prognosis in patients with hepatitis B-associated liver cirrhosis.

In this study, we found that Treg and Th17 cells in the peripheral mononuclear cells of the LC patients were negatively correlated. The imbalance of Treg/Th17 maybe was caused by increased IL-6 level in patients with hepatitis B-associated liver cirrhosis. IL-6 is a stimulating factor with a variety of biological activities and has an important role in regulating the balance between Treg and Th17 cells. When the body lacks IL-6, TGF-β induces the expression of FOXP3, which binds to RORγt and blocks its functions, leading to the transformation of naive T cells into Tregs. However, when IL-6 is present in the body, it acts with low concentrations of TGF-β to co-activate the STAT3 signaling pathway and removes the inhibition of RORγt by FOXP3, resulting in the transformation of naive T cells to Th17 cells [23] .

Elevated serum IL-6 levels have been observed in patients with hepatitis B-associated liver cirrhosis, which may be caused by the following mechanisms: (1) Overgrowth of intestinal bacteria leads to increased endotoxin production, and endotoxin induces the generation of IL-4, TNF and other immune complexes through a series of intracellular signaling transduction systems, all of which are IL-6 inducing agents. (2) Serious impairment of liver function occurs in liver cirrhosis, leading to weakened clearance of IL-6 by hepatic cells. Thus, the increased IL-6 levels in patients with liver cirrhosis lead to the transformation of naive T cells into Th17 cells and a reduced number of Treg cells and elevated number of Th17 cells, resulting in Treg/Th17 imbalance. A decrease in the number of Tregs further leads to reduced secretion of IL-10 and a weaker anti-fibrotic effect, while an increase in the number of Th17 cells further leads to increased IL-17 secretion and a profibrotic effect, resulting in aggravated liver fibrosis, promoting a vicious cycle.

Due to the limitations of the sample size in this study, the relationship between Treg and Th17 cells with the pathogenesis of hepatitis B-associated liver cirrhosis still needs to be explored further. For example, what factors influence the Treg/Th17 imbalance during the progression of hepatitis B-associated liver cirrhosis? In addition, we did not enroll hepatocellular carcinoma patient with hepatitis B-associated liver cirrhosis in this study, therefore, in hepatocellular carcinoma patients with hepatitis B-associated liver cirrhosis, what changes will appear in the expression of Treg and Th17? If the characteristics of this change are significantly different from those in patients with hepatitis B-associated liver cirrhosis without liver cancer, could the Treg/Th17 imbalance be an early-warning indicator for the progression of hepatitis B-associated liver cirrhosis to liver cancer? All these problems will be our research direction in the future.

Additionally,HBV-infection-associated liver diseases are a series of diseases, including chronic hepatitis B(CHB), liver cirrhosis(LC), hepatocellular Carcinoma(HCC). They also could be divided into non-cirrhotic hepatitis B diseases and cirrhotic hepatitis B diseases. In the present study, we found the significance of Treg/Th17 ratio with the patients of hepatitis B-associated liver cirrhosis, while Nan et al. [7] reported that Treg/Th17 imbalance was present in the disease of chronic hepatitis B. Therefore, it is important to explore new pathogenic mechanism of HBV-infection-associated liver disease and find better therapy through comparing Treg/Th17 ratio between non-cirrhotic Hepatitis B patients and cirrhotic Hepatitis B patients in the future study.

Another limitation is that the patients of hepatitis B-associated liver cirrhosis were not confirmed by biopsy. As we know, biopsy is a golden standard in diagnosing liver cirrhosis. In fact, it is hard to use in clinical experiences due to its invasion. Moreover, most of patients with liver cirrhosis deny the liver puncture biopsy for fear of the danger of bleeding,especially in the patients of decompensated liver cirrhosis with decreased platelets and coagulation factor. Therefore, the diagnosis of liver cirrhosis in clinical always come from causes(e.g., alcohol, hepatitis), symptoms(e.g., fatigue, loss of appetite, jaundice), blood test (e.g.,liver function, coagulation factor), imagnation test(e.g., Ultrasound, CT, or MRI scans). In the future study, if the patients would like to accept liver puncture biopsy, it is important to explore the significance of Treg/Th17 ratio between chronic hepatitis B patients with no cirrhosis and with cirrhosis.

In summary, we reported the imbalance of Treg/Th17 cells in patients with hepatitis B-associated liver cirrhosis. Treg/Th17 ratio was closely related to the clinical stage of hepatitis B-associated liver cirrhosis and might be used a promising indicator to discriminate CLC from DCLC. Therefore, we concluded that Treg/Th17 imbalance not only participated in the pathogenesis of hepatitis B-associated liver cirrhosis, but also associated with the progression and prognosis of hepatitis B-associated liver cirrhosis. In addition, large-scale, multicenter studies are needed to investigate the further association of Treg/Th17 imbalance and hepatitis B-associated liver cirrhosis, which might provide new therapeutic targets for hepatitis B-associated liver cirrhosis.

## Data Availability

All data generated or analyzed during this study are included in this published. article and its additional file.

## Abbreviations

ALB: Albumin
ALT: Alanine aminotransferase
CLC: Compensated liver cirrhosis
DCLC: Decompensated liver cirrhosis
HBV: Hepatitis B virus
LC: Liver cirrhosis
PT: Prothrombin Time
TBIL: Total bilirubin

## References

[1] Fattovich G (2003). Natural history and prognosis of hepatitis B. Semin Liver Dis.

[2] Vierling JM (2007). The immunology of hepatitis B. Clin Liver Dis..

[3] Zhu J, Yamane H, Paul WE (2010). Differentiation of effector CD4 T cell populations. Annu Rev Immunol.

[4] Romagnani S (2008). Human Th17 cells. Arthritis Res Ther.

[5] Kleinewietfeld M, Hafler DA (2013). The plasticity of human Treg and Th17 cells and its role in autoimmunity. Semin Immunol.

[6] Niu Y, Liu H, Yin D, Yi R, Chen T, Xue H (2011). The balance between intrahepatic IL-17(+) T cells and Foxp3(+) regulatory T cells plays an important role in HBV-related end-stage liver disease. BMC Immunol.

[7] Nan XP, Zhang Y, Yu HT, Sun RL, Peng MJ, Li Y (2012). Inhibition of viral replication downregulates CD4(+)CD25(high) regulatory T cells and programmed death-ligand 1 in chronic hepatitis B. Viral Immunol.

[8] Sato M, Suzuki S, Senoo H (2003). Hepatic stellate cells: unique characteristics in cell biology and phenotype. Cell Struct Funct.

[9] Lee UE, Friedman SL (2011). Mechanisms of hepatic fibrogenesis. Best Pract Res Clin Gastroenterol.

[10] Mannaerts I, Nuytten NR, Rogiers V, Vanderkerken K, van Grunsven LA, Geerts A (2010). Chronic administration of valproic acid inhibits activation of mouse hepatic stellate cells in vitro and in vivo. Hepatology.

[11] Wells RG (2008). Cellular sources of extracellular matrix in hepatic fibrosis. Clin Liver Dis.

[12] Jaffar Z, Ferrini ME, Girtsman TA, Roberts K (2009). Antigen-specific Treg regulate Th17-mediated lung neutrophilic inflammation, B-cell recruitment and polymeric IgA and IgM levels in the airways. Eur J Immunol.

[13] Bilate AM, Lafaille JJ (2012). Induced CD4+Foxp3+ regulatory T cells in immune tolerance. Annu Rev Immunol.

[14] Sakaguchi S (2011). Regulatory T cells: history and perspective. Methods Mol Biol.

[15] Claassen MA, de Knegt RJ, Tilanus HW, Janssen HL, Boonstra A (2010). Abundant numbers of regulatory T cells localize to the liver of chronic hepatitis C infected patients and limit the extent of fibrosis. J Hepatol.

[16] Zhang LJ, Zheng WD, Chen YX, Huang YH, Chen ZX, Zhang SJ (2007). Antifibrotic effects of interleukin-10 on experimental hepatic fibrosis. Hepatogastroenterology.

[17] Harrington LE, Hatton RD, Mangan PR, Turner H, Murphy TL, Murphy KM (2005). Interleukin 17-producing CD4+ effector T cells develop via a lineage distinct from the T helper type 1 and 2 lineages. Nat Immunol.

[18] O'Quinn DB, Palmer MT, Lee YK, Weaver CT (2008). Emergence of the Th17 pathway and its role in host defense. Adv Immunol.

[19] Sun HQ, Zhang JY, Zhang H, Zou ZS, Wang FS, Jia JH (2012). Increased Th17 cells contribute to disease progression in patients with HBV-associated liver cirrhosis. J Viral Hepat.

[20] Sparna T, Retey J, Schmich K, Albrecht U, Naumann K, Gretz N (2010). Genome-wide comparison between IL-17 and combined TNF-alpha/IL-17 induced genes in primary murine hepatocytes. BMC Genomics.

[21] Tan Z, Qian X, Jiang R, Liu Q, Wang Y, Chen C (2013). IL-17A plays a critical role in the pathogenesis of liver fibrosis through hepatic stellate cell activation. J Immunol.

[22] Meng F, Wang K, Aoyama T, Grivennikov SI, Paik Y, Scholten D (2012). Interleukin-17 signaling in inflammatory, Kupffer cells, and hepatic stellate cells exacerbates liver fibrosis in mice. Gastroenterology.

[23] Kimura A, Kishimoto T (2010). IL-6: regulator of Treg/Th17 balance. Eur J Immunol.

